# Human Gut-On-A-Chip Supports Polarized Infection of Coxsackie B1 Virus *In Vitro*

**DOI:** 10.1371/journal.pone.0169412

**Published:** 2017-02-01

**Authors:** Remi Villenave, Samantha Q. Wales, Tiama Hamkins-Indik, Efstathia Papafragkou, James C. Weaver, Thomas C. Ferrante, Anthony Bahinski, Christopher A. Elkins, Michael Kulka, Donald E. Ingber

**Affiliations:** 1 Wyss Institute for Biologically Inspired Engineering at Harvard University, Boston, Massachusetts, United States of America; 2 Molecular Virology Team, Division of Molecular Biology, Center for Food Safety and Applied Nutrition, U.S. Food and Drug Administration, Laurel, Maryland, United States of America; 3 Harvard John A. Paulson School of Engineering and Applied Sciences, Cambridge, Massachusetts, United States of America; 4 Vascular Biology Program, Boston Children’s Hospital and Harvard Medical School, Boston, Massachusetts, United States of America; University of Minnesota College of Veterinary Medicine, UNITED STATES

## Abstract

Analysis of enterovirus infection is difficult in animals because they express different virus receptors than humans, and static cell culture systems do not reproduce the physical complexity of the human intestinal epithelium. Here, using coxsackievirus B1 (CVB1) as a prototype enterovirus strain, we demonstrate that human enterovirus infection, replication and infectious virus production can be analyzed *in vitro* in a human Gut-on-a-Chip microfluidic device that supports culture of highly differentiated human villus intestinal epithelium under conditions of fluid flow and peristalsis-like motions. When CVB1 was introduced into the epithelium-lined intestinal lumen of the device, virions entered the epithelium, replicated inside the cells producing detectable cytopathic effects (CPEs), and both infectious virions and inflammatory cytokines were released in a polarized manner from the cell apex, as they could be detected in the effluent from the epithelial microchannel. When the virus was introduced via a basal route of infection (by inoculating virus into fluid flowing through a parallel lower ‘vascular’ channel separated from the epithelial channel by a porous membrane), significantly lower viral titers, decreased CPEs, and delayed caspase-3 activation were observed; however, cytokines continued to be secreted apically. The presence of continuous fluid flow through the epithelial lumen also resulted in production of a gradient of CPEs consistent with the flow direction. Thus, the human Gut-on-a-Chip may provide a suitable *in vitro* model for enteric virus infection and for investigating mechanisms of enterovirus pathogenesis.

## Introduction

Infections by human enteroviruses, which are small, non-enveloped, positive single-stranded RNA viruses that belong to the Picornaviridae family, are common in the United States and ubiquitous throughout the world [1,2]. Enteroviruses have been isolated from the lower and upper alimentary tracts, and transmission can occur by fecal-oral and respiratory routes depending on the species, strain, environmental conditions and type of illness. Replication at the primary site of infection (within gastrointestinal or respiratory epithelial cells) is usually followed by a viremic phase leading to infection of a secondary site [3]. With an estimated 10–15 million infections and at least 30,000–50,000 hospitalizations per year, enterovirus infections have been identified as a frequent cause of aseptic meningitis. Human enterovirus species also have been reported to contribute to development of fever, exanthemas, mild respiratory illness, bronchitis, pneumonia, encephalitis, poliomyelitis, myocarditis, and more recently, type 1 diabetes [2,4]. Development of model systems to study enterovirus infections is critical because there are no approved drugs or vaccines for treatment of members of this viral class, other than polio [2,3].

As a representative group of enteroviruses, coxsackievirus B (CVB) serotypes 1–6 are among the most studied pathogens within this group. In addition to causing fever, rash, and symptoms of an upper respiratory infection, CVB infections can cause myocarditis and target other organs, including pancreas and liver [1,2]. Recently, there has been an increase in severe neonatal illnesses and deaths due to Coxsackievirus B1 (CVB1) in the United States over other enterovirus serotypes, most likely due to increased incidence of multi-organ disease [5]. Studies aimed at targeting the initial site of replication (e.g., in the gastrointestinal epithelium) could have the most impact on reducing disease progression and severity. Enterovirus infection and replication are typically studied *in vitro* using established primate cell lines grown in monolayer; however, no single established cell line can support the growth of all cultivable enteroviruses [1]. Human intestinal cell lines grown in static culture also have been used to model enteric virus infection, however, these cultures are time-consuming to establish (21 days in culture), and they typically do not form villus structures or contain all four types of intestinal epithelial cells (absorptive, mucus-secreting, enteroendocrine, and Paneth cells), nor do they express genes associated with intestinal differentiation and function [6–9]. Alternatively, animal models can be applied to study CV infection *in vivo* but, in addition to obvious physiological differences between rodent and human intestinal immunity [10], they often present specific limitations. For instance, while murine CV-induced myocarditis models have been developed, virus inoculation was done via the intraperitoneal route bypassing the native initial site of virus infection in the gastrointestinal epithelium [11]. In addition, the human CVB1 receptor and associated co-receptors that are essential for infection of polarized epithelial cells are not present in mice, so given that the molecular basis for CVB1 infection is different in mice compared to humans, the clinical relevance of results obtained with these models is unclear [12,13].

Some human enterovirus strains (e.g., CV serotype A strains) remain difficult to grow in culture, while others undergo rapid replication, produce infectious virions and induce cytopathic effects (CPEs) when they infect permissive cell lines [1,14,15]. For example, CVB-1 infection of cell cultures results in detectable increases in positive and negative strand synthesis, as well as capsid proteins, within a few hours following infection. Assembly and production of infectious virus progeny are rapid and followed by cytolysis, prefaced by characteristic CPEs, with the time of their appearance being dependent on the number of infectious particles in the culture, or the ‘multiplicity of infection’ (MOI). Typically, the higher the MOI, the more rapidly CPEs occur, beginning in less than 24 hours in cultured primate cells [2,15,16]. Several studies also have sought to understand and define the molecular mechanisms by which CVBs enter, infect, and kill human intestinal epithelial cells using immortalized Caco2 or HT29 intestinal cell lines [7–9]. While these models have advanced our understanding of CVB’s interaction with intestinal cells, the intestinal cell monolayers used in these studies fail to exhibit high levels of differentiation, and do not provide the structural complexity of the specialized villus epithelium found in living intestine. In addition, all of these past *in vitro* studies were carried out under static conditions that do not recapitulate the dynamic physical environment of the human intestine that experiences peristaltic-like mechanical motions and intraluminal fluid flow, which are known to contribute significantly to intestinal physiology. As a result, much remains unknown regarding the specific molecular events involved in virus replication and release from infected human intestinal epithelium. This can also be said of human enteric viruses in general which includes species/strain classified within a very broad and genetically diverse range of virus families, including Caliciviruses (e.g., human noroviruses, human sapoviruses), Astroviruses (e.g., human astrovirus), Reoviruses (e.g., human rotaviruses, Hepevirus (e.g., human hepatitis E virus) and other Picornaviruses (e.g., hepatitis A virus). In fact, many of these viruses are difficult to culture in standard culture systems, while others remain non-cultureable in any *in vitro* model tested.

To begin to address this pressing need for new *in vitro* models of enteric virus infection, we investigated whether a recently developed human Gut-on-a-Chip microfluidic device that permits culture of human intestinal epithelium under conditions that mimic the physical microenvironment of human intestine, including physiological fluid flow and cyclic peristalsis-like mechanical deformations, and that exhibits high levels of intestinal differentiation *in vitro* [17,18] can provide this function. In contrast to static cultures, when human Caco-2 intestinal epithelial cells are grown in this device under these mechanically active conditions, they spontaneously form three-dimensional (3D) villi-like structures containing proliferative crypts and lined by absorptive, mucus-secreting, and enteroendocrine and Paneth cells [18], and exhibit transcriptome profiles [19] that more closely replicate the microenvironment of the human small intestine which is a primary site of enteric virus infection. In addition, as the Gut-on-a-Chip contains a second lower ‘vascular’ channel separated from the epithelial channel by a porous extracellular matrix-coated membrane, it offers the ability to study polarized infection of the villus epithelium when virus is introduced either apically through the intestinal lumen or basally via the vascular compartment, and to collect produced virus continuously under flow. Thus, the focus of the present study was to evaluate the potential of the human Gut-on-a-Chip as a model of enteric virus infection using CVB1 and evaluating development of CPEs over time, as well as measuring infectious virus production from apical and basal effluents, cytokine production, and caspase activation rather than to directly compare CVB1 infection of the Gut-on-a-Chip to well characterized static cell culture systems [6–8].

## Materials and Methods

### Human Gut-on-a-Chip microdevice

The Gut-on-a-Chip devices containing two hollow microchannels (each 1 mm wide x 200 μm tall x 1.4 cm long) separated by a porous membrane (10 μm diameter circular pores with a 25 μm spacing) were fabricated using polydimethylsiloxane (PDMS; Sylgard, Corning) with soft lithography techniques, as previously reported [17,18]. The inner channels and porous membrane within the device were plasma activated, functionalized with (3-Aminopropyl)trimethoxysilane (Sigma, St. Louis, MO) and coated with type I collagen (30 μg/mL; Gibco) and Matrigel (100 μg/mL; BD Biosciences, Bedford, MA) prior to cell plating. Human Caco2 intestinal cells (Caco2 BBE, Harvard Digestive Disease Center) were suspended in Dulbecco’s Modified Eagle Medium (DMEM; Gibco, Grand Island, NY) supplemented with 10% fetal bovine serum (FBS; Gibco), 100 U/mL penicillin, and 100 μg/mL streptomycin. The cells were then seeded (1.5x10^5^ cells/cm^2^) into the top channel of the device and grown statically on the matrix-coated porous membrane overnight before initiating flow (30 μL/hr; shear stress = 0.02 dyne/cm^2^) through both the top and bottom channels for 6 days; when mature, each gut chip contained ~3 to 4x10^5^ cells. Cyclic suction was applied through parallel chambers to rhythmically stretch (10% strain; 0.15 Hz) and relax the porous membrane and adherent cells starting on day 2 to mimic peristalsis-like deformations. Intestinal permeability was measured by flowing Inulin (4 Kda)-FITC (100μg/mL in medium) at 60μL/hr through the top channel for 24h and measuring the fluorescence intensity of medium flowing out of the top and bottom channels in 3 individual gut chips. The apparent permeability was calculated using the following formula:

$$P_{app}=\frac{J}{A\cdot \Delta C}$$

With *P*_*app*_ = Apparent permeability, *J* = Molecular flux, *A* = Total area of diffusion, and *ΔC* = Average gradient (~1 due to the low flow rate).

### Virus infection

Coxsackievirus B1 (Conn-5 strain, ATCC VR-28) was obtained from ATCC (Manassas, VA) and high-titered stocks were produced as described by Kulka et al [16] and aliquoted for use in the current study. Six days after seeding, 5 μl of stock virus (or media, for uninfected chips) was added to each chip and allowed to adsorb (incubated without flow) for two hours. At this time, the virus was removed from the chips and washed with additional media to remove any unbound virus. Cells were then placed back under flow and stretch conditions for the duration of the infection. Apical and basal effluents were sampled at 6, 24 and 48 h post infection (hpi).

### RNA extraction/RT-qPCR

We used one-step RT-qPCR on extracted samples to track onset of virus replication in post-infection effluents as well as a means of estimating dilutions for subsequent analyses by plaque assay. Adsorption media and effluents (collected apically or basally) (140 or 280 μl) were extracted with the QIAamp Viral mini RNA kit (QIAGEN, Valencia, CA) per manufacturer’s instructions. Briefly, samples were incubated with lysis buffer containing carrier RNA, mixed with ethanol to precipitate the viral RNA, subjected to QIAamp mini column binding, wash and elution. Five μl of eluate was subjected to one step RT-qPCR, on 96 well plates using the Quantitect Probe PCR kit (Qiagen) with PanEv primers and probes [20,21] on the ABI 7500 FAST (Life Technologies). Primers and FAM-labeled probe were manufactured by Integrated DNA Technologies (Coralville, IA). The following conditions were used for one-step RT-qPCR reactions: an initial RT step for 30 minutes at 50°C followed by a denaturation step at 95°C for 15 minutes, followed by 45 cycles of 95°C for 15 seconds, 58°C for 45 seconds, 72°C for 30 seconds. Known titers of CVB1 stock viral RNA (previously determined by plaque assay) were serially diluted and used to generate a standard curve to estimate viral load.

### Plaque assay

FRhK4 cells were seeded for confluence in 8 mm dishes and were infected with appropriate dilutions of the virus samples in DPBS. Specifically, 500 μl aliquots of dilutions were inoculated in duplicate onto monolayers for adsorption at 37°C, 1 h followed by removal of the inoculum. Plates were then overlaid with MEM medium supplemented with 2% serum and 0.5% agarose (Sigma, St. Louis, MO) and incubated at 37°C in 5% CO_2_ for 48 h. A second overlay was added on the infected cells consisting of 0.5% agar and 2% neutral red. Plaques were then observed at 3-5h and manually counted. Countable plates were those having 5 to 50 plaque forming units (PFU) per well. Because CVB1 infectious titer can decrease with repeated freeze-thaw cycles and over time (personal observation), the stock virus titer was re-determined by plaque assay along with the samples collected in order to get a more accurate gauge on the number of infectious virus particles added to each chip for each experiment (“input virus”). In order to normalize the data between experiments and individual chips, the value of CVB1 titer at each time point (6, 24, 48 hpi) is calculated as the average of the total number of plaque forming units (pfu) in the effluent at the time of collection (“output virus”) divided by the “input virus” for that experiment (n = 3 experiments), shown as fold-change from input. It is important to note that values at 24 and 48 h pi are reflective of virus collected from 6–24 hpi and 24–48 hpi, respectively.

### Microscopic analysis

For immunofluorescence microscopy, gut chips were washed with PBS, fixed in 4% paraformaldehyde, permeabilized with 0.2% Triton-X (Sigma, USA), blocked with 1% bovine Serum Albumin (Sigma, USA) and incubated for 1 h at 37°C with antibodies against CVB1 (1:100, MAB944; Millipore, USA), Villin (1:100, Abcam, USA) and/or F-Actin (Phalloidin-Alexa-568, Molecular Probes, USA); nuclei were counterstained with DAPI. Fluorescence imaging was carried out using confocal laser scanning microscopy (SP5 X MP DMI-6000, Leica, Germany) and image processing and 3-dimensional Z-stack reconstruction was done using Imaris (Bitplane, Switzerland) and ImageJ (http://imagej.nih.gov/ij/) software. To carry out scanning electron microscopy, gut chips were used in which the top channel was not irreversibly bonded to the membrane, which permitted the device to be dismantled without perturbing the cultured cells. After 7 days of culture, cells were fixed in 2.5% glutaraldehyde (Electron Microscopy, USA), treated with 0.5% osmium tetroxide (Sigma) followed by dehydration in graded ethanol washes. Cells were then dried in a critical point dryer (Tousimis Auto Samdri 815 Series A, Tousimis, Rockville, MD), sputter coated with gold and imaged with a scanning electron microscope (Tescan, Czech Republic).

### Caspase assay

For the caspase Western blotting studies, cells cultured in the gut chips were trypsinized, pelleted, extracted for total protein with RIPA buffer, run on SDS-PAGE, transferred to nitrocellulose, and Western blots were performed with antibodies to caspase-3 (Cell Signaling Technology, Danvers, MA) and actin (Developmental Studies Hybridoma Bank, Iowa City, IA) as previously described [16]. Secondary IRDye antibodies were purchased from Li-Cor Biosciences (Lincoln, NE) and the blot was scanned on the Li-Cor Odyssey scanner.

### Cytokine analysis

Apical and basal effluents of flowing medium were collected and analyzed for IP-10 and IL-8 at 24 hpi using a custom Magplex assay kit (Millipore, USA). Concentration analysis was determined according to the manufacturer’s instructions, using a Luminex FlexMap 3D system coupled with a Luminex XPONENT software (Luminex, USA).

### Statistical analysis

All results and error bars are presented as mean + standard error of the mean (SEM). Data were analyzed with an unpaired Student’s t test using Graphpad Prism (GraphPad Software Inc., San Diego, CA, USA). Differences between groups were considered statistically significant when p<0.05 (*p<0.05, **p<0.01, ***p<0.001).

## Results

### CVB1 infection of the human Gut-on-a-Chip

**Fig 1:**
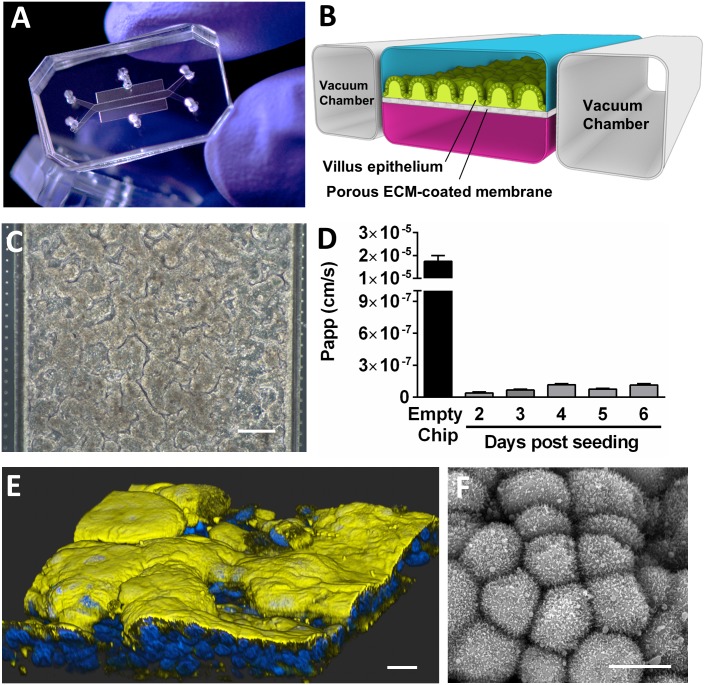
Human Gut-on-a-Chip microfluidic culture device. Photograph (**A**) and schematic diagram (**B**) of the human Gut-on-a-Chip device. (**C**) Phase contrast micrograph of human Caco-2 intestinal epithelial cells cultured for 6 days in a Gut-on-a-Chip under apical flow (30μl/hr; 0.02 dyne.cm^-2^) and cyclic mechanical strain (10% at 0.15 Hz); bar, 100 μm. (**D**) Apparent permeability (P_app_) of the epithelium assessed by adding fluorescent inulin-FITC daily to the upper channel for 6 days after seeding (n = 3 chips). Note that an ECM-coated Gut-on-a-Chip without cells used as a control exhibited a very high permeability. (**E**) Confocal immunofluorescence micrograph of human villus intestinal epithelium formed inside the Gut-on-a-Chip and stained for villin (yellow) to visualize the apical brush border and nuclei (blue); bar, 10 μm. (**F**) Scanning electron micrograph of the apical surface of the villus epithelium cultured for 6 days in the Gut-on-a-Chip under flow and mechanical strain. Note the microvilli at the surface of the cells (bar, 10 μm).

The microfluidic Gut-on-a-Chip is composed of a clear, flexible PDMS polymer that contains two central hollow microchannels separated by a porous matrix-coated membrane, with hollow, full-height, vacuum chambers located on either side that are used to apply cyclic stretching forces to the cultured epithelium to mimic peristalsis like motions (Fig 1A and 1B). Consistent with previous studies [17,18], when human Caco2 intestinal epithelial cells were cultured on the matrix coated porous membrane in the top channel for 6 days under continuous perfusion (~0.02 dyne cm^-2^) and cyclic mechanical strain (10%; 0.2Hz), mimicking fluid flow and physiological peristaltic motions of the human intestine respectively [22,23], the cultured epithelium cells spontaneously formed undulating villus-like structures (Fig 1C) that exhibited a tight epithelial barrier (low apparent permeability) when analyzed with fluorescent inulin (Fig 1D). Immunofluorescence microscopy (Fig 1E and S1 Movie) and scanning electron microscopic analysis (Fig 1F) confirmed that these villus-like structures were ~50 to 100 μm tall and that the polarized epithelial cells exhibited villin-containing microvilli along their apical surfaces.

**Fig 2:**
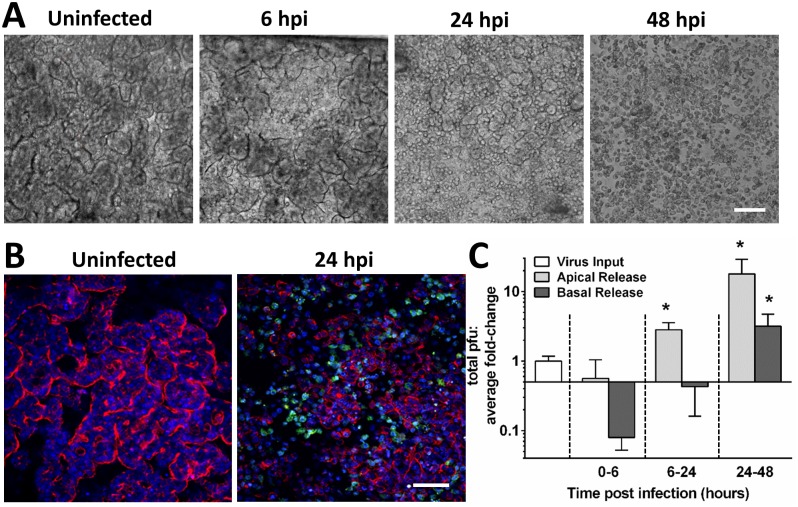
Coxsackie virus B1 readily infects the human Gut-on-a-Chip. (**A**) Phase contrast micrographs of the villus epithelium within the Gut-on-a-Chip apically infected with CVB1 at 6, 24 and 48h post infection (hpi); bar, 100 μm. (**B**) Confocal immunofluorescence micrographs of uninfected and apically infected villus epithelium 24hpi that were stained for CVB1 (green), F-actin (red) and nuclei (blue). Note the destruction of villi in infected samples; bar, 100 μm. (**C**) Graph showing quantitation of viral loads in the effluents of the apical (epithelial) versus basal (vascular) microchannels after apical infection of the gut chips with CVB1 at an MOI of 0.2 (*p < 0.05 compared to 0–6 hpi).

To determine the ability of CVB1 to infect the human Gut-on-a-Chip, eight separate chips were inoculated apically through the lumen of the epithelial channel (MOI = 0.2) and incubated at 37°C for 2h; the cultures were then perfused with medium and infected devices were monitored for 48h. While no CPE was evident at early times (6h) when compared to uninfected controls, loss of villus morphology indicative of epithelial injury, along with cell rounding and detachment, were detected by 24 hours post infection (hpi) (Fig 2A). By 48 hpi, villi could no longer be detected and the integrity of the entire epithelium was lost in infected chips (Fig 2A), indicative of a sudden complete loss of barrier function. Immunofluorescence staining for F-actin and CVB1 24hpi confirmed the loss of villus epithelial structures and the prevalence of CVB1-containing cells inside the infected devices (Fig 2B).

### CVB1 virions replicate and are preferentially released apically

Quantification of CVB1 virions by plaque assay in samples collected from effluents taken from the apical and basal microchannels revealed an increase of up to 30 fold in virus titer over time (1 x10^5^ to 3 x 10^6^ pfu), confirming that CVB1 viruses underwent active replication and released infectious virus particles in the human Gut-on-a-Chip (Fig 2C). Interestingly, we detected significantly (p< 0.05) greater numbers of virions in the effluent from the apical compared to basal microchannel at 24 and 48 hpi, indicating that there was polarized release of the newly formed virions from the apical or luminal membrane of the human intestinal epithelium.

**Fig 3:**
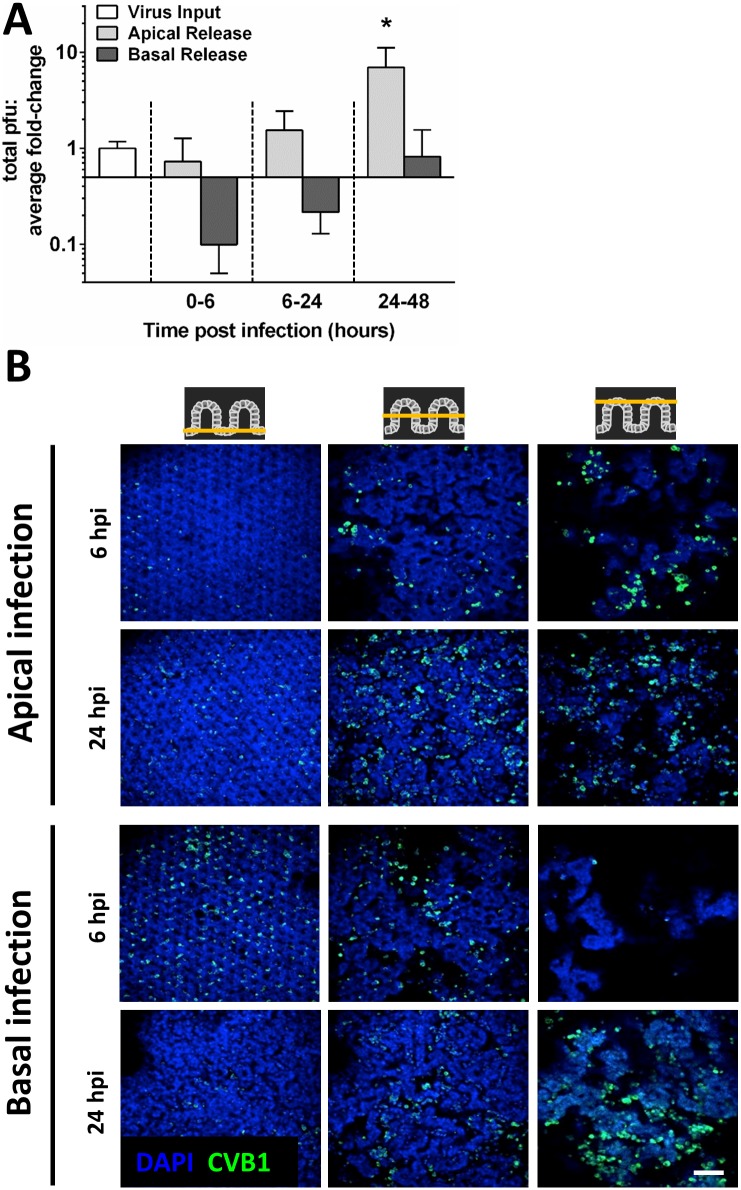
CVB1 release is polarized towards the epithelial lumen. (**A**) Graph showing quantitation of viral loads in the effluents of the apical (epithelial) versus basal (vascular) microchannels after basal infection of the gut chips with CVB1 at an MOI of 0.2 (*p < 0.05 compared to 0–6 hpi). (**B**) Confocal fluorescence micrographs of apically and basally infected gut chips at 6 and 24 hpi, showing horizontal sections at the base, middle and top of the villi (left to right columns). Infected chips were stained for CVB1 (green) and nuclei (blue); bar, 100 μm.

To investigate whether the apical release of CVB1 was a direct consequence of the apical route of infection, eight individual gut chips were infected basally using the same MOI (0.2) and infection conditions described above. Virus titration by plaque assay revealed that CVB1 release was again preferentially directed towards the apical side of the epithelium (Fig 3A), thus, demonstrating selective polarized release of CVB1 virions from the luminal surface of this villus intestinal epithelium. Additionally, CVB1 titration demonstrated that basal inoculation induced less virus release than apical inoculation (Fig 3A, 3B and S1 Fig). Interestingly, confocal imaging of the apically infected Gut Chips at three different horizontal planes revealed that CVB1 virions were preferentially localized in the top half (apical) regions of the villi structures at 6 and 24 hpi (Fig 3B). In contrast, the viruses were primarily localized at the base of the villi at 6 hpi in basally infected cultures (Fig 3B); however, they progressively moved to the apical portions of the villi by 24 hpi (Fig 3B). These results are consistent with directional transport of the infectious virions (post-replication and assembly), and suggest that intracellular transport and secretion of enteroviruses may be polarized towards the cell apex in the villus gut epithelium.

### Effects of luminal flow on infection propagation

**Fig 4:**
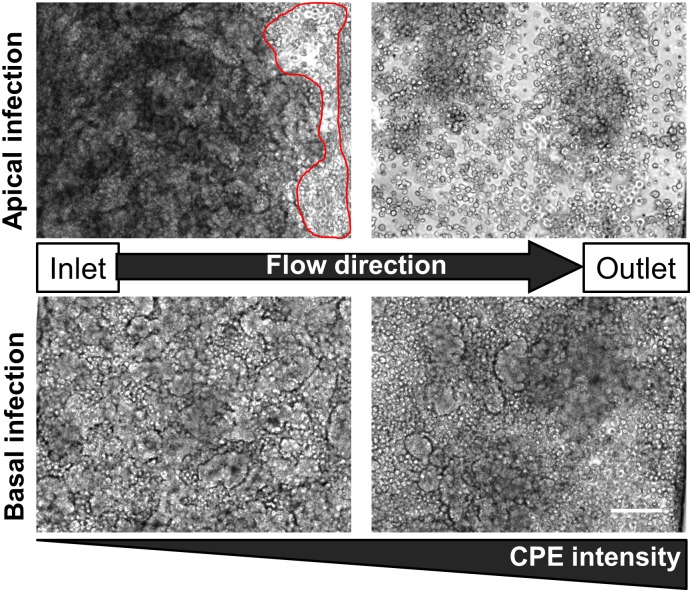
Apical fluid flow generates a gradient of CPE. Phase contrast micrographs of apically and basally CVB1-infected Gut-on-a-Chips at 24hpi. The flow direction (from the inlet to the outlet) is from left to right (the arrow). Note the increasing CPE intensity towards the downstream outlet; bar, 100 μm.

Because we applied continuous flow with a physiologically relevant level of shear stress above and below the intestinal epithelium [22,23] in this study, we were also able to analyze the effects of these dynamic conditions on viral infection. Interestingly, we observed a gradient of CPEs across the epithelium when it was infected with CVB1 under these conditions, ranging from low levels of infection near the inlet to high levels of CPE towards the outlet. While large villus destruction with only a few rounded rare remaining adherent cells were observed in the downstream region of the infected chips close to the outlet, only small localized CPEs were detected in upstream areas (Fig 4). Furthermore, when the chips were infected basally, we observed a delay in cytopathic response compared to the apically infected chips (Fig 4), and it required 48 hpi for the basally infected chips to display a similar level of epithelial injury as the apically infected chips exhibited at 24 hpi (S2 Fig). Again, these results are consistent with a lag in CPE formation due to the time required for trafficking of virions from basal to apical side of the epithelium.

### Polarized cytokine release and apoptosis in the infected epithelium

**Fig 5:**
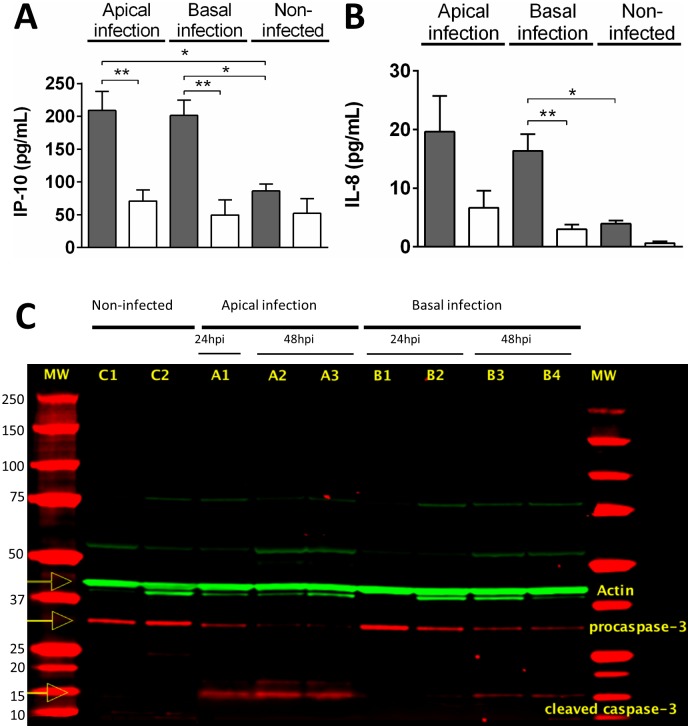
Cytokine secretion and caspase-3 expression in infected gut chips. IP-10 (**A**) and IL-8 (**B**) were quantified in effluents from the apical (gray bars) and basal (white bars) microchannels in gut chips that were either uninfected or infected apically or basally (n = 4 chips/condition; *p < 0.05, **p < 0.001) at 24 hpi. (**C**) Epithelium lining gut chips that were uninfected (controls C1 and C2), or infected apically (A1, A2, A3) or basally (B1, B2, B3, B4), or infected apically or basally, were lysed and pro-caspase-3 and cleaved caspase-3 levels were visualized on the gel as indicated.

To further characterize the interaction between CVB1 and the Gut-on-a-Chip, we analyzed the concentration of pro-inflammatory cytokines IP-10 and IL-8 released into the basal and apical effluents at 24 hpi (Fig 5A and 5B). Both cytokines levels were significantly elevated in the infected chips compared to negative controls regardless of whether the virus was inoculated apically or basally; however, IP-10 and IL-8 levels were consistently much higher in the apical effluent, despite the loss of cell integrity which would allow for relatively unhindered passage of the cytokines from the apical to basal compartment. The data suggest a polarized release of cytokines to the luminal side of the epithelium independent of the route of virus entry. Finally, as infection resulted in extensive loss of epithelial integrity suggestive of considerable cell death, we analyzed apoptosis levels in these cultures by measuring levels of caspase-3 activation (Fig 5C). As expected, pro-caspase 3 was partially cleaved within 24 hpi, and almost completely by 48 hpi in apically infected cultures. In contrast, cleavage was negligible at 24 hpi in basally infected samples although it was evident by 48 hpi, consistent with a delayed infection when cultures when infected basally.

## Discussion

CVs are responsible for a broad range of diseases that can be life-threatening in children and neonates; however, little is known about their induced pathogenicity and how they interact with the human gut epithelium, their primary site of infection. While animal models have been used to study the biology and pathogenesis of CV infection [24,25], current murine models largely rely on inoculation into the intraperitoneal space or directly into the brain, and thus, they fail to recapitulate the natural route of infection through the respiratory and intestinal mucosa [26–28]. Recently, a 3D culture model that recapitulates shear stress in the intestine by culturing Caco2 cells on extracellular matrix-coated beads in a rotating wall vessel bioreactor was used to study CVB3 infection [29]. While this system demonstrated further differentiation of Caco2 cells than a static system, it did not support induction of villi-like structures nor does it allow basolateral sampling like the Gut-on-a-Chip. Additionally, this culture system lacks the ability to incorporate other cell types and still requires a minimum of 2–3 weeks to induce the observed differentiation, as opposed to 5–6 days for the Gut-on-a-Chip device. In contrast, the Gut-on-a-Chip microfluidic culture system provides an *in vitro* investigative tool to study enterovirus infection, intracellular replication, and polarized release of new virions in human villus intestinal epithelium in an environment that permits continuous collection of produced virions under flow, and it also supports co-culture of the intestinal epithelium with living microbiome [18,19]. We found that CVB1 can efficiently infect polarized intestinal epithelial cells when inoculated on either their apical and basolateral surfaces; however, we observed a polarized trafficking of virions and cytokine production following infection in this human intestinal epithelium model.

Apical release of virions is a common feature of viruses infecting the respiratory and intestinal epithelium (e.g., Sendai virus, hPIV1, hPIV3, Respiratory Syncytial Virus, Influenza, Measles, Mumps, Hepatitis A), highlighting the strong need for an *in vitro* model of human intestinal mucosa that can recapitulate this form of polarized infection observed *in vivo* to permit future mechanistic analysis [30–37]. In the present study, we showed that we could mimic this apical trafficking of CVB1 virions to the intestinal lumen *in vitro* regardless of the infection route (basal or apical). While an ascending “escalator” mechanism could play a role in the host defense arsenal to prevent virus access to the submucosal tissue and entry into the blood stream, the usual outcome of enteric virus infection is viremia and infection of a secondary tissue [1–3,5,15]. While we cannot exclude the possibility of passively “traveling” through to the basal side, the Gut-on-a-chip forms a permeability barrier that effectively resists transport of small fluorescent molecules [17,19], and so our results suggest that CVB1 entry into the bloodstream may occur *in vivo* in regions where the epithelial barrier is destroyed secondary to apoptosis or via transport by infected immune cells. Alternatively, the presence of low levels of infectious virus in the effluent of the basal vascular microchannel in this study may indicate that lower amounts of virus are released or shed from the basal surface of the epithelium, which could then either infect the endothelial barrier or cross into the bloodstream by active or passive transport [38].

The significant induction of release of the cytokine IP-10 in the CVB1-infected Gut-on-a-Chip is consistent with *in vivo* mechanisms of RNA detection occurring during picornavirus infection and replication. TLR3-mediated intracellular detection of CVB1 double stranded RNA produced during the viral replicative cycle induces the production of Type I interferon which, in turn, triggers secretion of IP-10 [39]. The antiviral effect of IP-10 against CVB3 has been demonstrated in mouse cardiomyocytes through recruitment of CD4+, and CD8+ T cells as well as NK cells [40]. Consistent with our findings, a previous study reported an increase of IP-10 and IL-8 mRNA levels in another type of cultured human intestinal cell (HT-29 cells) when infected with a related CV (CVB3) [41]. Similar induction of IL-8 secretion also has been reported in CVB5-infected human bronchial epithelial cells [42], CVB4-infected human pancreatic endothelial cells [43] and CVB3-infected human myocardial fibroblasts [44]. We recently showed that LPS stimulation of the Gut-on-a-Chip co-cultured with human PBMCs resulted in a release of IL-8 polarized to the basolateral compartment, as observed *in vivo* [19]. In contrast, we show here that IL-8 is released apically, suggesting that enterovirus infection may induce a specific reversal of this directionality. Further studies would be necessary to investigate this mechanism *in vivo*.

Our results showed that apoptotic signaling mediated by capase-3 activation is activated in human intestinal epithelium in response to CVB1 infection of the Gut-on-a-Chip, which is consistent with previous reports of CVB1-infected FRhK4, Vero cells, HEK293 cells, and CV-1 cells [45]. While others have reported a calcium-dependent, non-apoptotic mode of cell death in polarized Caco-2 cells infected with CVB [9], our results implicate caspase-3 as a player in the virus-mediated CPEs we observed in this study. The difference observed between these two studies may be due to the fact we used CVB1, rather than CVB3. The past study also used a 5- to -25-fold higher MOI and investigated caspase activation at a much earlier time (5 hpi), which is actually consistent with our results as we did not observe significant CPEs or caspase activation until 24 or 48 hpi, depending on the route of infection. However, we did not examine the effect of pan-capase inhibitor on induction of CPEs and therefore cannot exclude the possibility that CVB1-induced cell destruction may also be occurring by a caspase-independent apoptotic mechanism as has been reported for several CVB serotypes including CVB1 in other model systems (31). Regardless of the actual mechanism involved, our ability to detect cytokine response to infection, virus replication, infectious virus production, progressive apoptosis, and epithelial destruction at a low MOI is likely more relevant for natural CVB infections where the numbers of infectious particles present on food are presumably very low. By leveraging the capacity of the Gut-on-a-Chip to incorporate commensal microbes in contact with the human intestinal epithelial cells, future studies may better recapitulate infection of the human intestine than previous culture models. Additionally, our model of polarized epithelial infection has the ability to differentiate between an apical and a basolateral route of infection, as well as determining if there is a preference for which subpopulation of epithelial cell gets infected, which may prove to be important for understanding enteric virus replication and infection propagation in the future.

Virus inoculation and subsequent infection of static cultures as done in past studies can result in multiple rounds of infection of the same cell monolayer, resulting in complete destruction of the entire epithelium [7,8]. In contrast, using this microfluidic model with continuous flow, we were able to detect a gradient of CPEs and villus destruction that correlated with the flow direction, suggesting that newly-formed virions released from cells near the inlet traveled with the flow and infected downstream cells, hence modeling secondary infections and disease propagation, which is not possible with static culture models. Interestingly, when we initiated these studies, we presumed that the constant flow would remove any newly-produced virions and thus reduce previously observed infection rates. However, instead we observed high CVB1 replication efficiencies in the Gut-on-a-Chip, which resulted in almost complete destruction of the villi within 24 hours after infection. Thus, this model may provide an ideal platform with which to study other enteric viruses that have historically been difficult or impossible to culture, including human norovirus.

Because they are spread via the fecal-oral route, enteric viruses remain a public concern and potential hazard with regard to food safety issues. While human norovirus and hepatitis A virus are currently identified as the viruses most frequently involved in outbreaks of foodborne illness, more than half of foodborne illnesses of suspected microbiological origin are unidentified due in part to the absence of diagnostic confirmation of virus identity and *in vitro* human cell models [46]. Given the presence of a large suspected, but currently unknown and unidentified viral burden, it is likely that non-hepatitis A, non-norovirus enteric viruses are contributors to this burden. Therefore, development of a broadly applicable *in vitro* model for human enteric infection would be important for understanding their infectivity, molecular biology, and pathophysiology, as well as developing mitigation strategies and treatments for viruses responsible for food borne illness and human infection. Recently, substantial efforts have been made to develop such models [47] and the novel human Gut-on-a-Chip *in vitro* culture system described here may provide a functionally unique approach to the study of a broad range human enteric viruses; and therefore, an alternative and/or adjunct to current human and non-human model systems.

## Supporting Information

S1 Fig Graph comparing quantification of viral loads in the effluent of the apical (epithelial) microchannel after apical vs basal infection of the gut chips with CVB1 at an MOI of 0.2. (TIF)

S2 Fig Phase contrast micrograph of basally CVB1-infected Gut-on-a-Chips at 48hpi. (TIF)

S1 Movie A movie showing a confocal 3D reconstitution of Caco-2 cells cultured inside a Gut-on-a-Chip for 6 days under flow and cyclic mechanical stretch and stained for Villin (yellow), Klf4 (red). Nuclei were counterstained with DAPI (blue). Bar, 20 μm.
(AVI)
