# Supplementary figures and images for: Human Gut-On-A-Chip Supports Polarized Infection of Coxsackie B1 Virus In Vitro
Source: PLoS One. 2017 Feb 1;12(2):e0169412. doi: 10.1371/journal.pone.0169412 (PMC5287454; doi:10.1371/journal.pone.0169412)

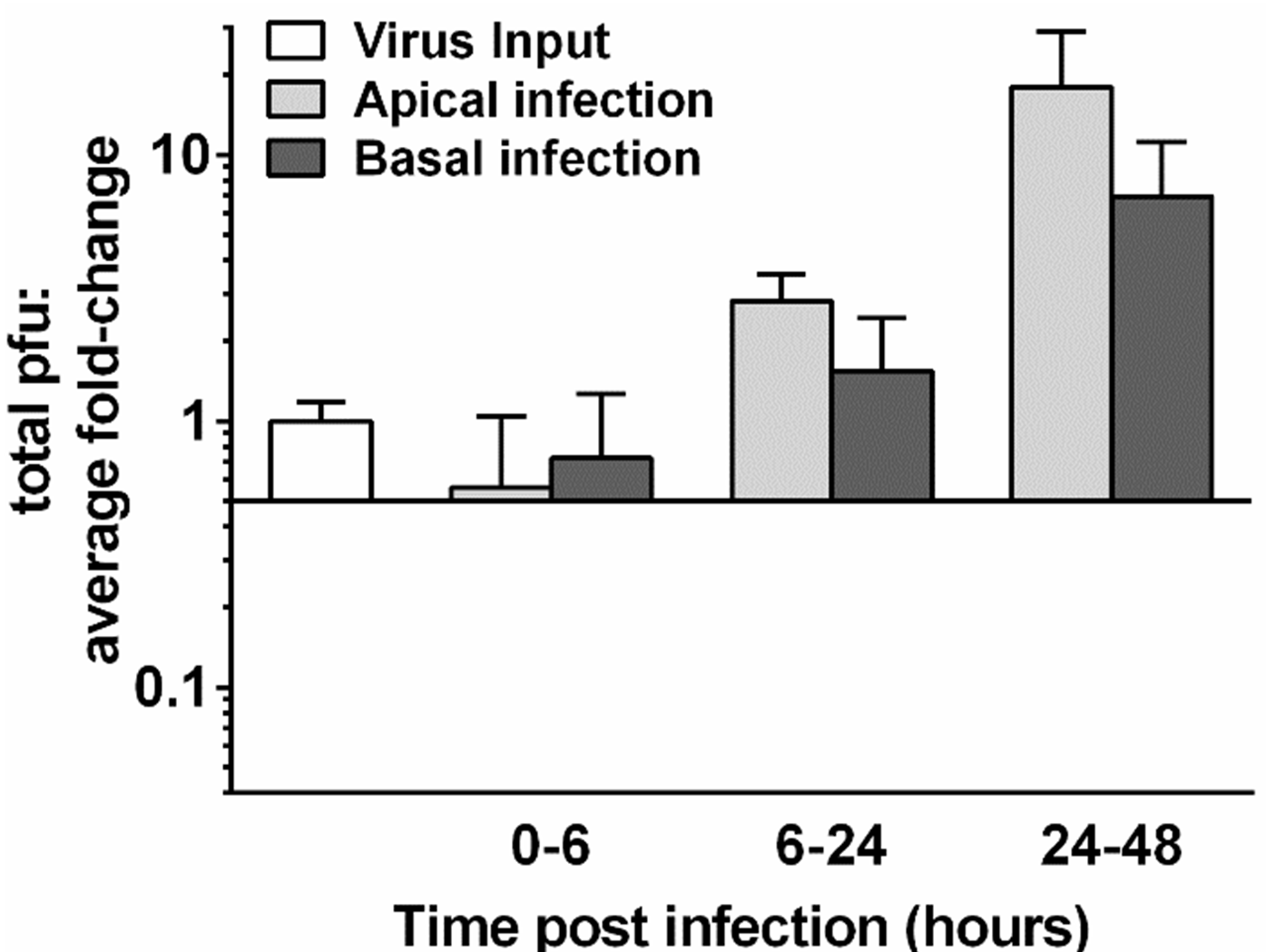

Supplement: S1 Fig — (TIF) [file pone.0169412.s001.tif]

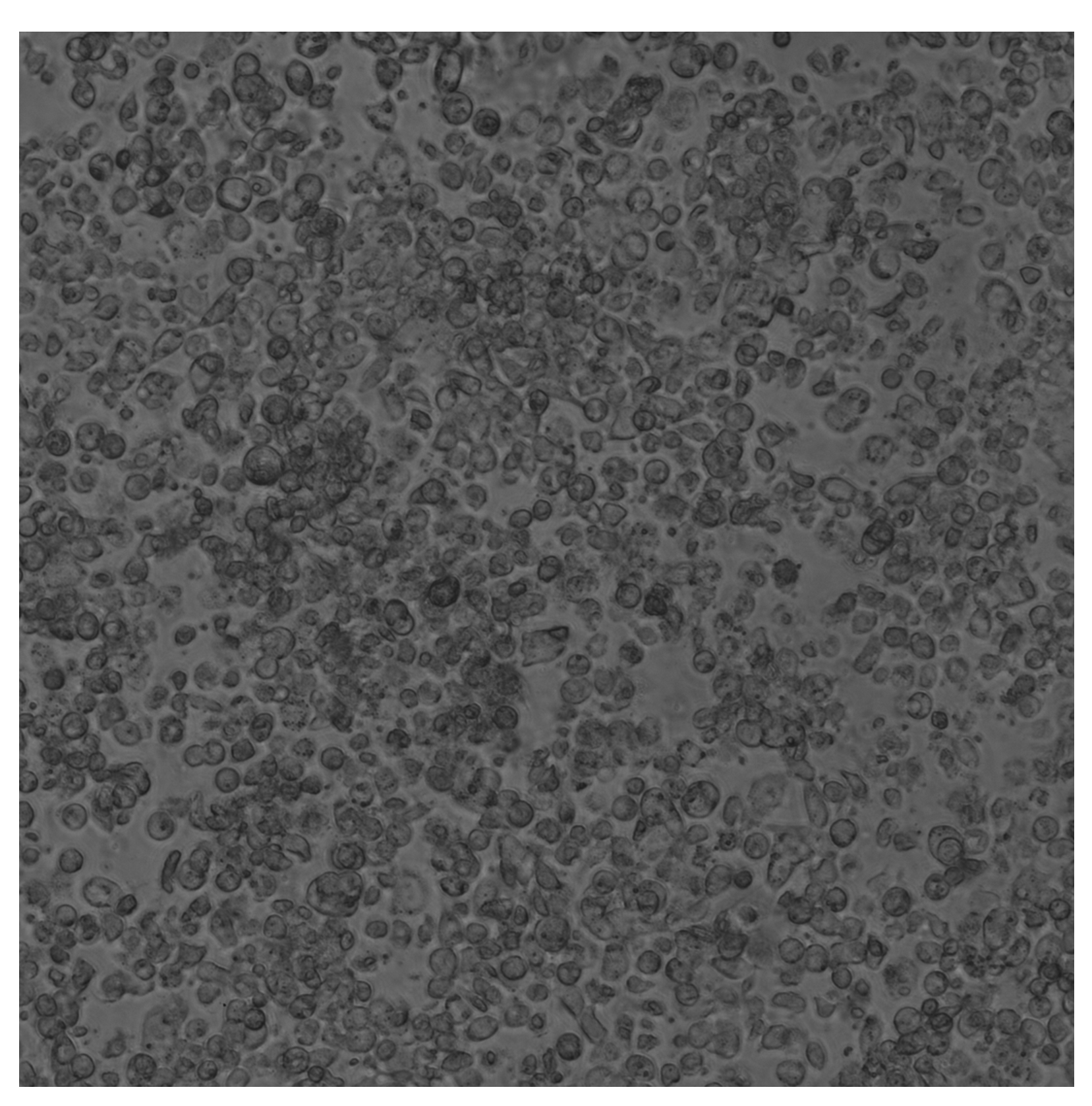

Supplement: S2 Fig — (TIF) [file pone.0169412.s002.tif]
